# Ecological and Human Health Risk Assessment of Heavy Metals in Cultured Shrimp and Aquaculture Sludge

**DOI:** 10.3390/toxics10040175

**Published:** 2022-04-02

**Authors:** Salma Sultana, Mohammad Belal Hossain, Tasrina R. Choudhury, Jimmy Yu, Md. Sohel Rana, Mohammad Abu Noman, M. Mozammal Hosen, Bilal Ahamad Paray, Takaomi Arai

**Affiliations:** 1 Department of Fisheries and Marine Science, Noakhali Science and Technology University, Noakhali 3814, Bangladesh; salma0212@student.nstu.edu.bd (S.S.); rana0212@student.nstu.edu.bd (M.S.R.); 2 School of Engineering and Built Environment, Griffith University, Brisbane, QLD 4111, Australia; jimmy.yu@griffith.edu.au; 3 Chemistry Division, Atomic Energy Centre Dhaka (AECD), Bangladesh Atomic Energy Commission, Dhaka 1000, Bangladesh; m.mozammalhosen@gmail.com; 4 State Key Laboratory of Biogeology and Environmental Geology, China University of Geosciences (Wuhan), Wuhan 430074, China; abu.noman.nstu@gmail.com; 5 Department of Zoology, College of Science, King Saud University, P.O. Box 2455, Riyadh 11451, Saudi Arabia; bparay@ksu.edu.sa; 6 Environmental and Life Sciences Programme, Faculty of Science, Universiti Brunei Darussalam, Jalan Tungku Link, Gadong BE1410, Brunei Darussalam; takaomi.arai@ubd.edu.bn

**Keywords:** heavy metals, shrimp culture, aquaculture sludge, human health risks

## Abstract

Shrimp is one of the major export products in South Asian countries and also an eminent source of nutrition for humans. Hence, any negative effect of this industry may affect not only the country’s economy but also human health. Therefore, in this study, we aimed to assess heavy metal contamination and associated human health risks in cultured shrimp (*Penaeus monodon*) and aquaculture sludge collected from three shrimp farms of the Cox’s Bazar district, Bangladesh. The results showed that among the eight metals studied, Pb (17.75 ± 1.5 mg/kg) and Cu (9.43 ± 2.8 mg/kg) levels in all shrimp samples were higher than the recommended limit, whereas the concentrations of Cd (0.09 ± 0.03 mg/kg), Mn (4.83 ± 2.2 mg/kg), As (0.04 ± 0.02 mg/kg), Hg (0.02 ± 0.006 mg/kg), Zn (18.89 ± 2.9 mg/kg) and Cr (0.69 ± 0.6 mg/kg) were within the permissible level. The concentrations of Mn (1043.37 ± 59.8 mg/kg), Cr (30.38 ± 2.1 mg/kg), Zn (74.72 ± 1.13 mg/kg) and Cu (31.14 ± 1.4 mg/kg) in the sludge of all farms were higher than the recommended limit, whereas the concentrations of Pb (20.23 ± 1.9 mg/kg), Cd (0.09 ± 0.2 mg/kg), As (0.44 ± 0.34 mg/kg) and Hg (0.08 ± 0.02 mg/kg) in all sludge samples were lower than the threshold limits. However, the estimated daily intake (EDI), targeted hazard quotient (THQ) and hazard index (HI) assessed for potential human health risk implications suggested that Pb and Cr may pose non-carcinogenic health effects, although carcinogenic risks (CR) values were acceptable for consumers. However, the pollution load index (PLI) of the studied area was below 1, which indicates low deterioration of the area. Geoaccumulation index (Igeo) and contamination factor (CF) analyses revealed that study area is unpolluted and sludge is enriched with metals in the following order: Mn > Zn > Cu > Cr > Cd > Hg > Pb > As.

## 1. Introduction

As a result of rapid economic advancement and industrial development, heavy metal contamination has become more severe and one of the major environmental problems globally [1,2]. Furthermore, heavy metal discharge from agricultural intensification and waste disposal are increasing in aquatic environments, posing a threat to invertebrates, fish and humans [1,3]. When heavy metals are consumed in significant amounts or allowed to accumulate above a certain threshold, they prompt arbitrary binding with cell biomolecules, such as chemicals and proteins, resulting in compounds that can affect their metabolisms [4]. Heavy metal contamination is not only a threat to aquatic organisms, but it also poses a serious health risk to humans [2]. When heavy metals and metalloids are present in large concentrations, they become hazardous to all living things, including humans. For example, an excessive amount of Hg, As, Pb and Cd contaminants can be harmful to living organisms, and long-term exposure can cause illness or death [5]. Hg, on the other hand, is one of the most toxic metals in the environment. Methyl mercury contamination causes inhibition of protein synthesis, microtubule disruption, an increase in intracellular Ca^2+^ and a disruption in synapse function. Long-term Cd consumption has also been associated with injuries such as prostatic proliferation, cellular breakdown in the lungs, bone fractures and renal failures, even at a low dosage of roughly 1 mg/kg [6,7]. An excessive amount of Pb can harm humans [8], causing sensory system problems, cerebral impairment, skeletal hematopoietic capacity problems and even death [9]. Excess Cr has been demonstrated to be carcinogenic to human health [10,11]. However, investigations have showed that the majority of these heavy metals accumulate in various organs of aquatic organisms, which are widely seen as markers of health issues for both animals and people who consume seafood [1,12,13]. This requires careful consideration and deliberate efforts on a national and global scale.

Fish and shellfish are regarded as the most common bioindicators for pollutants because they occupy a higher level in the aquatic food chain [14]. Furthermore, they have been used as a key source of protein by humans. As a result, humans are extremely sensitive to high heavy metal concentrations [15]. As a result, analysing heavy metal concentrations in shrimp could be a useful tool for determining the impact of anthropogenic pressure on both the ecosystem and human health. Aquaculture sludge, on the other hand, serves as essential sinks or repositories for heavy metals and is also recognised as a critical component in determining the level of metal toxicity in farmed areas [16].

Shrimp aquaculture has flourished predominantly in low-lying tropical and subtropical coastal locations due to the suitable environment and availability of space. It has grown dramatically in Bangladesh in the last two decades and the production climbed from 1.60 million metric tonnes (MT) in 2002–2003 to 2.41 million MT in 2019–2020. During 2019–2020, roughly 1.27 lakh MT of shrimp were produced through farming, accounting for 45.14 percent of the country’s total aquaculture production [17]. However, *Penaeus monodon* alone contributes 22.88 % of the country’s aquaculture production, followed by *Macrobrachium rosenbergii* (18.08 percent) [17]. As a result, the southeastern and southwestern parts of Bangladesh have a large number of shrimp hatcheries and farms. In Cox’s Bazar, for example, there are currently 57 shrimp hatcheries in operation [18]. Cox’s Bazar is the southernmost section of Bangladesh, located 150 kilometres south of Chittagong Port, and is home to a variety of businesses, such as shipbreaking yards, paint, glass, synthetic substances, manures, and petrochemical industries [19]. As a result, farming areas in Cox’s bazar are extremely susceptible to heavy metal contamination.

Several studies have looked into heavy metal contamination and pollution load estimation in shrimp from the island of Saint Martin [11,20], Khuln-Satkhira Region [21], and Buriganga River [11] in Bangladesh, whereas very few studies have examined heavy metals in farmed shrimp species [22]. Unfortunately, published research concerning heavy metal contamination and associated health risks through consumption of shrimp from the southeast coast of Bangladesh is scant, and no information is available for aquaculture sludge. Therefore, in the present study, we aimed to assess the levels of heavy metals in farmed shrimp species (*Penaeus monodon*) and aquaculture sludge from three different shrimp farms in the Cox’s Bazar region of Bangladesh and to estimate the potential environmental and human health risks.

## 2. Materials and Methods

### 2.1. Study Area

**Figure 1:**
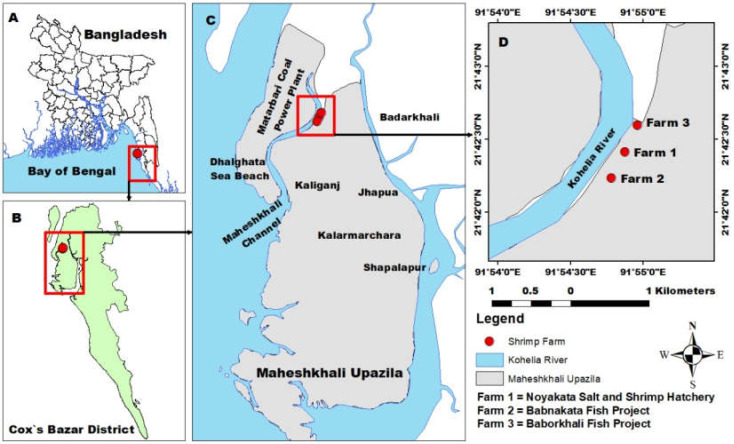
Sampling locations of shrimp and aquaculture sludge from Cox’s Bazar, Bangladesh. ((**A**)—Map of Bangladesh, (**B**)—the Cox’s Bazar district, (**C**)—Maheshkhali Upazilla, (**D**)—Sampling points near the Kohelia River).

The study was carried out in the commercial shrimp farms located at Maheshkhali Upazila of Cox’s Bazar district, Bangladesh. Samples (shrimp and sludge) were collected from three different commercial shrimp farms, namely Noyakata Salt and Shrimp Hatchery (21°70′6943″ N to 91°91′4561″ E), Babnakata Fish Project (21°70′3906″ N to 91°91′3027″ E) and Baborkhali Fish Project (21°71′0773″ N to 91°91′6004″ E), all of which are located along a river, named the Kohelia River (Figure 1). 

### 2.2. Sample Collection

From August to October 2020, thirty samples were collected to analyse eight heavy metals (Pb, Cd, Cr, Mn, Cu, Zn, As and Hg). Five shrimp specimens and five sludge samples were taken from each farm in order to establish homogeneity. Sludge samples were collected by a mud corer at a depth of 0–10 cm of the farm bed. Each sample was a composite of three grabs to average the heterogeneity of sludge, and approximately 200 g of sludge was collected from each farm. Traditional fishing nets were used to collect shrimp samples. All samples were immediately transferred to sealed zipper bags and stored in the refrigerator until further analysis.

### 2.3. Sample Preparation, Digestion and Metal Extraction

After transfer to the laboratory, all the shrimp samples were cleaned with deionized water, and the muscle tissue of each sample was extracted and cut into smaller pieces. The extracted muscle tissues were air dried after washing with deionized water to eliminate extra moisture. Subsequently, the samples were homogenized with a food processor, and 200 g of sample was stored at −20 °C for test purposes. Later on, two grams of the homogenized muscle tissue was digested with a polytetrafluoroethylene digestion vessel. along with the 6 mL of supra-pure nitric acid. At the same time, the samples were digested through a two-step temperature program with a microwave oven (MARS’5 XP-1500 plus, CEM Corporation, Matthews, NC, USA) in which the rotating magnetron had a maximum power of 1600 W. In the initial step, the temperature was raised to 180 °C for 10 min and maintained at that temperature for 15 min during the subsequent step. After digestion, the samples were cooled to room temperature, and the solutions were diluted to a final volume of 20 mL by adding deionized water.

A portion of the representative sludge samples was placed on petri dish and in a hot air oven at a temperature of 110 °C for 8 h. After cooling, the samples were ground with a mortar and pestle. Then, the ground samples were sieved through a 2 mm mesh strainer and homogenized. One gram of dehydrated and powdered sludge samples was put into a pre-acid-washed 100-mL beaker, and 15 mL of aqua regia (mixture of HNO_3_ and HCl, optimally in a molar ratio of 1:3) was added. At last, the beaker was set into a hot plate at 100 °C temperature covering through a watch glass, and heating continued as long as brown fumes were released. The solution was evaporated through heating without boiling. When the digestion was finished, to decrease the total volume of substance, the maximum amount of sludge samples was dissolved in acid. After cooling, the solution was filtered through a membrane filter (125-mm) (Whatman^®^ Schleicher & Schuell, Darmstadt, Germany), and transferred into a 25 mL volumetric flask by adding deionized water. After that, the digested samples were stored at 4 °C until analysis of metals.

### 2.4. Analysis of Samples and Quality Assurance

All the samples, along with the sample blanks (only deionized water) and standards, were aspirated into a flame atomic absorption spectrophotometer (AAS) (model: Varian AA240FS & AA280 Z, Varian Inc., Palo Alto, CA, USA). A calibration curve was executed for concentration vs. absorbance. Through the least square method and using the fitting of a straight line, the data were statistically analysed. While calculating the concentration of various elements, necessary corrections were made considering the blank samples. Replicate samples, blank samples and certified reference materials (CRM) were used for the accuracy of the experiment. Oyster tissue samples from the National Institute of Standards and Technology, as well as TORT-2 samples from the National Research Council Canada, were used as the reference materials. Recovery results indicated good accuracy and precision, as they were within 10% of the certified values (Table S1).

### 2.5. Health Risk Assessment of Shrimp

#### 2.5.1. Estimated Daily Intake (EDI)

The estimated daily intake (EDI) of the metals were calculated using the metal concentration, daily food intake and body weight of the consumer and calculated using the following formula [10,23]:

EDI=(DFC × MC)/BW

where DFC is the amount of food (fish) consumption per day, and MC is the mean metal concentration in shrimp muscle tissue. Based on the “Report of the household income and expenditure survey 2015”, in this study, we considered an average of 49.5 g of daily fish intake for a Bangladeshi adult person (60 kg).

#### 2.5.2. Target Hazard Quotient (THQ) 

The target hazard quotient (THQ) is the measure of non-carcinogenic risk due to contaminant exposure. The THQ was calculated using the following equation [24]:

$$\mathrm{THQ}=\frac{\mathrm{MC}\;\times \;\mathrm{IR}\;\times \;\mathrm{EF}\;\times \;\mathrm{ED}\;\times \;\mathrm{CF}}{\mathrm{RfD}\;\times \;\mathrm{BW}\;\times \;\mathrm{ATn}}\times 10^{-3}$$

where MC stands for the metal concentration in shrimp muscle tissue (mg/kg dw); fish ingestion rate is defined as IR (49.5 g/kg dw) [25]; EF means the frequency of the exposure (365 days/year); ED means the duration of exposure (30 years); CF defines the conversion factor, 0.208; RfD is the oral reference dose of heavy metals (we considered the RfD as 0.2 mg/kg for Pb, 0.005 mg/kg for Cd and 0.009 mg/kg for Cr) [24]; the average body weight of an adult person is defined with BW (70 kg); and the average exposure time is defined with ATn, which is 10,950 days for non-carcinogens [24,26].

#### 2.5.3. Hazard Index (HI)

The hazard index (HI) is the sum of the THQ by multiple elements (Pb, Cd and Cr) [24,26].

$$\mathrm{HI}=\overset{n}{\underset{i=k}{\sum }}\mathrm{THQ}$$

where THQ represents the risk-factor-estimated individual metals. The HI value over 10 depicts high non-carcinogenic risk effects for its consumers. 

#### 2.5.4. Carcinogenic Risk (CR)

Carcinogenic risk (CR) denotes the possibility of developing cancer of an individual over his/her lifetime because of exposure to a potential carcinogen. Carcinogenic potency slope factor (CSF) is needed to estimate the cancer risk, which is found for very few metals (As, Cr, Cd and Pb) by USEPA [27]. The CR was calculated as follows [27,28]:

CR = CSF × EDI

where CSF stands for the carcinogenic potency slope factor, which is 0.0085 (mg/kg/day) for Pb, 6.3 for Cd, 0.5 for Cr and 1.5 for As, as provided by USPEA [29]; EDI stands for the estimated daily intake of heavy metals; and CRs ranging from 10^−4^ to 10^−6^ are acceptable, whereas more than 10^−4^ depicts probability of developing cancer over a human lifetime. 

### 2.6. Risk Assessment in Aquaculture Sludge

#### 2.6.1. Geoaccumulation Index (Igeo)

The geoaccumulation index (Igeo) is a tool for assessing the environmental contamination state by comparing it to geochemical background concentrations. [30]. Geoaccumulation index (Igeo) for metals was determined using the following expression [30]:

Igeo = log_2_ (C_n_/1.5B_n_)

where C_n_ means the concentration of the studied heavy metals in the sludge; B_n_ stands for the geochemical background value of a given metal, which was taken from Turekian and Wedapohl [31] because they provided background values for every kind of sediment, and in our region, such values are not well established; and the factor 1.5 is used to account for the probable variation in the background values [30] classified Igeo values into seven grades or classes (Table S2).

#### 2.6.2. Contamination Factor (CF)

Contamination by metal in the sludge is expressed in terms of a contamination factor (CF) and calculated as follows:

$$\mathrm{CF}=\frac{C_{n\;\mathrm{sample}}}{B_{n}\;\mathrm{shale}}$$

where C_n sample_ depicts the metal concentration in the sludge sample, and B_n_ stands for the geochemical background value of the given metal [31]. Hakanson [32] has provided four grade ratings of sediments based on CF values (Table S2).

#### 2.6.3. Pollution Load Index (PLI)

Pollution load index (PLI) is an integrated approach to assess the sludge quality, including all metals, such as Cr, Ni, Cu, As, Cd and Pb [33]. PLI is defined as the nth root of the multiplications of the contamination factor (CF) of metals [33].

$$\mathrm{PLI}=({\mathrm{CF}}_{1}\times {\mathrm{CF}}_{2}\times {\mathrm{CF}}_{3}\times \ldots {\mathrm{CF}}_{n}{)}^{1/n}$$

where CF_metals_ depicts the ratio of the concentration of each metal to the background values in sludge—in short, CF_metals_ = CH_metal_/CH_back_. A PLI value > 1 means polluted, whereas <1 indicates no pollution [34].

### 2.7. Statistical Analysis

Correlation matrices based on Pearson’s correlation, the most used multivariate statistical technique, were used to measure the relationships among heavy metals in the sludge samples. Principal component analysis (PCA) was performed to determine the associations of metals in shrimp and sludge. Hierarchical cluster analysis (HCA) based on Ward’s method was performed to identify the grouping of metals in tissue and sludge. The dataset was log-transformed before the multivariate analyses, and the statistical methods were performed with a 95% confidence interval (significance *p* < 0.05). The statistical analysis was carried out using free statistical software, PAST (version 3.0). Additionally, the site map was tailored by Arc GIS (v. 10.3) software, and other graphical representations were performed using GraphPad (version 7) and Origin pro (2015).

## 3. Results and Discussion

### 3.1. Heavy Metal Concentration in Shrimp 

A wide range of heavy metal concentrations were observed in the examined shrimp specimens. The concentrations of eight selected heavy metals (Pb, Cd, Cr, Mn, Cu, Zn, As and Hg) in shrimp muscles maintained the following hierarchy: Zn (18.89 ± 1.5 mg/kg) > Pb (17.75 ± 1.5 mg/kg) > Cu (9.43 ± 2.8 mg/kg) > Mn (4.83 ± 2.2 mg/kg) > Cr (0.69 ± 0.6 mg/kg) >Cd (0.09 ± 0.004 mg/kg) > As (0.04 ± 0.02 mg/kg) > Hg (0.02 ± 0.006 mg/kg) (Table 1).

In our study, the average concentration of Zn (Zn 18.89 ± 1.5 mg/kg; wet weight) was higher than that of other metals in the shrimp muscle (Table 1). However, Zn concentration was less than the permissible limit in the shrimp muscle tissue. Zn is an essential micronutrient for living creatures, as it is a catalyst for nearly 300 enzymes in all aquatic organisms. Therefore, a relatively high level of Zn is required to balance certain biological functions. Zn is involved in most of metabolic pathways in humans, and its insufficiency can lead to loss of appetite, inhibition of growth, skin changes and immunological abnormalities [43]. However, excessive Zn consumption can cause acute negative effects [44,45]. The higher concentration of Zn than that of other metals—although lower than the threshold concentration—meets the needs of shrimp muscle tissue without making it harmful for consumption.

The average concentrations of Pb in shrimp muscle in our study was 17.75 ± 1.5 mg/kg wet weight. The European Union advises that the tolerance level of Pb in crustacean is 0.5 mg/kg (wet weight) [35]. The maximum allowable concentration of Pb contaminants in shellfish is 1 mg/kg [46]. However, our analysis of shrimp tissues from commercial farm of Cox’s Bazar region revealed that the mean concentration of Pb was much higher than the recommended limit. The concentration of Pb in shrimp was 35.5 times higher than the maximum permissible limit for crustacean in Bangladesh and the European Union (Table 1). Pb is a non-essential component; neurotoxicity, nephrotoxicity and many other negative health consequences may be caused by contamination [47]. Sarkar et al. reported that Pb concentration in shrimp tissue from Satkhira, Bangladesh ranged from 0.54 to 1.16 mg/kg [21], and Ahmed et al. reported that Pb concentration in *M. rosenbergii* from the Buriganga River was 0.51 mg/kg [11]. Although the reported contaminations were higher than the permissible guideline values, we found much higher levels of contamination than those reported above. However, shrimp species such as *M. rosenbergii* and *P. monodon* are bottom-dwelling organisms almost always in contact with sediments; hence, the sediments could be the major sources of Pb contamination [21].

In the present study, the concentration of Cd in shrimp muscle tissue was 0.09 ± 0.03 mg/kg. A maximum Cd level of 0.5 mg/kg in shrimp is recommended by the European Community legislation [35], and our reported findings are far below the guideline values (Table 1). Cd is an element able to produce chronic toxicity even if present at a low concentration. Cd naturally exists in the environment in tiny concentrations; the concentration in the aquatic environment may increase as a result of industrial processes, such as smelting or electroplating and the addition of fertilizers. Excess Cd may cause renal failure and the softening of bones as a result of long-term or high-dose exposure contamination [48] and prostate cancer in response to high levels of Cd [49]. However, Cd was not the major contaminant in our study, as the concentration of Cd was lower than the standard limit, and previously reported concentrations in Buriganga River and Saint Martin Island in Bangladesh; Sabah in North Boreno; and the Gangetic Delta, and Red Sea in Saudi Arabia (Table 1). 

The mean concentration of chromium obtained was 0.69 ± 0.6 mg/kg (wet weight) in the shrimp tissue, whereas the recommended tolerance level of Cr in crustacean is 0.5 mg/kg according to European Commission [35] and 1 mg/kg in Bangladesh [39]. In Bangladesh, tannery and poultry waste is utilized as fish feed, which may contribute to excessive Cr contamination in shrimp. Sarker et al. found the Cr concentration in the muscle of *Penaeus monodon* was 0.68 mg/kg, which is a similar level to that we obtained in our study [21]. Another previous study found the average Cr concentration was 1.59 ± 0.9 mg/kg in the Buriganga River, which is higher than our reported value, whereas the finding from Saint Martin Island was much lower than our observed value (Table 1).

In terms of Mn, the average concentrations (4.83 ± 2.2 mg/kg) in all shrimp samples were higher than those previously reported but lower than the threshold limit. According to TFC [37] the threshold level of Mn in crustacean is 20 mg/kg. A similar type of study was conducted by Abdel-Baki et al. [50] in Saint Martin Island, Bangladesh, and reported that the concentration of Mn in shrimp muscle was <0.2 mg/kg, whereas we obtained 24 times higher concentrations of Mn in shrimp muscle tissue. However, in Buriganga River, the recorded Mn concentration was 35.25 ± 1.48 mg/kg, which is much higher than our finding (Table 1). However, some species, such as mollusks, sponges and diatoms have higher ability to accumulate Mn in their tissues. However, an excess of or deficiency in Mn concentration can cause negative health consequences [51]. Severe skeletal and reproductive abnormalities were detected in mammals due to Mn scarcity [52].

Our study revealed that the average concentration of Cu in shrimp tissue was 9.43 ± 2.8 mg/kg. Cu concentrations in food should not surpass the value of 20.0 mg/kg (wet weight) according to the UK Food Standards Committee Report [53]. According to Bangladesh and the EU tolerance level, the permissible guideline concentration of Cu is 5 mg/kg in crustaceans. Therefore, the Cu level in shrimp muscle in our study was higher than the guideline values of the EU and Bangladesh but lower than the FAO recommended guideline [54]. A high intake of Cu can cause negative health impacts, such as liver and kidney damage [44], although the optimum concentration is necessary for good health. However, consumption of contaminated fish may result in increased Cu concentration in humans through the food cycle. In Buriganga River and Saint Martin Island of Bangladesh, the average Cu concentrations were found to be 575.34 ± 61.8 and 5.049 ± 0.07, respectively (Table 1). The Cu concentration in our study was lower than that found in the Buriganga River but higher than that reported for Saint Martin Island (Table 1). Similarly, our reported concentration is lower than the Cu concentration reported in Gangetic Delta but higher than that reported for the Red Sea, Saudi Arabia (Table 1).

In comparison with other metals, the concentrations of As (0.04 ± 0.02 mg/kg) and Hg (0.02 ± 0.006 mg/kg) were the lowest in our study. Ingestion of seafood is the major pathway of As and Hg exposure to humans [_ENREF_5055]. Hg and As are potential environmental and public health problems, but in our study, both of their concentrations were well below the permissible limit. Similarly, in the Persian Gulf, the concentrations of Hg (500 µg/kg) and arsenic (6000 µg/kg) in giant tiger shrimp (*Penaeus semisulcatus*) were well below the maximum permissible levels [55]. However, among the numerous chemical forms of As, the organic form is less harmful than the inorganic form. It is difficult to know which types of arsenic are present and how to quantify them properly. Approximately 10% of the total arsenic is to be determined as inorganic arsenic [56]. According to recent studies, extremely low concentrations of As may perform as an endocrine disruptor [57], whereas prolonged exposure to inorganic As may lead to several health difficulties, including in the gastrointestinal tract, respiratory tract, skin, liver, cardiovascular system, hematopoietic system and nervous system [58]. Seafood contains a large amount of organic As [59,60], and without transformation, it could be discharged in urine easily and quickly [59]. Hg is a trace element found mostly in hazardous organic form (methylmercury, MeHg+), which constitute 75–90% of the total Hg present in fish [61]. However, several previous studies also reported that the concentrations of As and Hg were below the guideline values in fish and shrimp along the Bay of Bengal coastline [20].

### 3.2. Heavy Metal Concentration in Sludge

**Figure 2:**
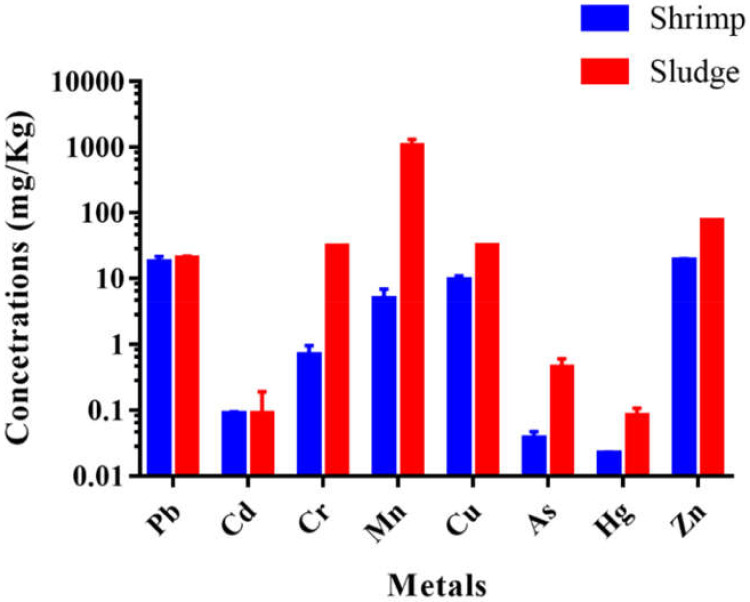
Comparison of metal concentration in examined shrimp muscles and aquaculture sludge in the present study.

The variation of different heavy metals in aquaculture sludge from commercial shrimp farms of Cox’s Bazar region is represented in Table 2. The analysis of the data determined the following order of heavy metal accumulation in sludge: Mn > Zn> Cu > Cr > Pb > As > Cd > Hg. The data indicated that Mn was highly accumulated in the sludge, whereas Hg was least concentrated (Figure 2). The heavy metal concentrations were compared with TRV (toxicity reference value) and LEL (lowest effect level), which represent the concentration below which adverse effects are rarely expected. The sediment quality guidelines (SQGs) represent the concentration above adverse effects are expected to occur frequently, as well as the tolerance level that signifies permissible metal levels (Table 2). In the present study, concentrations of Mn exceeded all the well-recognized standard values. The concentration of Zn ranked second highest in the sludge samples and surpassed some standard criteria. The concentration of Cr also exceeded some standard levels, but the rest of the metals in the sludge were well below the threshold level (Table 2). However, in comparison with previous studies, all heavy metal concentrations in our study were higher than the reported values in the sediment of Feni River Estuary, whereas most of the metals, except As and Zn, had higher concentrations than those reported in the Sangu River Estuary (Table 2). 

### 3.3. Public Health Risk for Shrimp Consumption

#### 3.3.1. Estimated Daily Intake (EDI)

**Figure 3:**
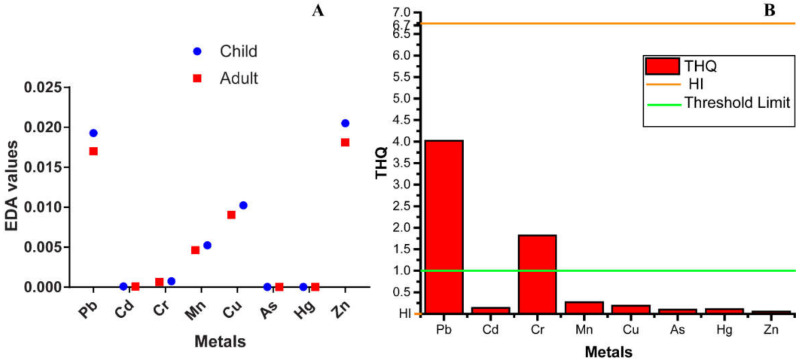
Public-health-related hazard indices ((**A**)-EDA, (**B**)-THQ & HI) for metal concentrations in shrimp.

The EDI of selected toxic heavy metals from shrimp consumption by the average coastal adults of Bangladesh is presented in Figure 3. EDI, based on the oral reference dose (RfD) for an individual element [74], reflects the daily exposure to the toxic element and is executed to avoid any harmful effect on human health. The recommended daily allowance (RDA) of the elements Pb, Cd, Cr, Mn, Cu, As, Hg and Zn was set by the WHO [75] as 0.25, 0.07, 0.23, 2–5, 0.9, 0.15, 0.04 and 11 mg/kg/ person, respectively. The estimated EDI of the people was compared with RDA, denoting that mean EDI values of the metals were lower than RDAs. The values lower than RDA guidelines suggest a lower possible health effect of the elements on consumers. However, it would not be wise to take it as a permanent measurement to reach a final conclusion [50].

#### 3.3.2. Target Hazard Quotient (THQ) and Hazard Index (HI) 

If the THQ < 1, the exposed individual is unlikely to experience adverse health effects; for THQ ≥ 1, there could be a likelihood of possible health hazards [26]. Similarly, the HI results also followed the THQ trend. The hazard index (HI) from THQs is denoted as the total of the hazard quotients [24]. In the present study, the THQ values of Pb and Cr were higher than the threshold (>1), which indicates potential health hazards through Pb and Cr contamination. When the HI value is lower than 1, it is considered safe for human consumption, but when HI > 1, it could be hazardous [55]. In our study, the combined risk factor of all examined metals (HI) was much higher than the acceptable limit (>1) in shrimp species (Figure 3). Therefore, continuous and/or excessive consumption of shrimp species could result in a chronic non-carcinogenic health effect. Although the assessment of THQ and HI for human health risk evaluation has no dose response relation to the examined elements [76], humans can dramatically suffer in the long run due to multiple simultaneous pollutants [77].

#### 3.3.3. Carcinogenic Health Hazard

Target lifetime carcinogenic risk (TR) was assessed for Cd, Cr, As and Pb due to exposure from shrimp consumption. The TR values for Cd, Cr, As and Pb from shrimp consumption were 6.1 × 10^−4^, 3.7 × 10^−4^, 3.7 × 10^−4^ and 1.6 × 10^−4^, respectively. In general, the estimated carcinogenic risk factor (TR) is categorized based on three criteria. Estimated cancer risks lower than 10^−6^ are considered to be negligible, above 10^−4^ are unacceptable and risk values between 10^−4^ and 10^−6^ are generally considered an acceptable range [29,76]. However, in our study, the carcinogenic risk value of Cd, Cr, As and Pb were within the acceptable range (Figure 3). Hence, carcinogenic risks are not likely to occur as a result of heavy metals through consumption of shrimp. However, this status is not guaranteed to remain stable, as metal bioaccumulation of the aquatic species may increase with exposure time and varies with size, age, food and feeding habitat. 

### 3.4. Environmental Health Concerns about Heavy Metals in Sludge

Geoaccumulation index (I_geo_), contamination factor (CF) and pollution load index (PLI) were estimated to evaluate the status of environmental health hazards related to metal concentration in sludge. Among them, I_geo_ values were used to explain sludge quality and degree of heavy metal contamination [78]. The index of geoaccumulation (Igeo) shows that aquaculture sludge collected from different shrimp farms of Cox’s Bazar is not polluted with any of the examined heavy metals, as the I_geo_ values are <0. However, CF values of Pb, Cd, Cu, As, Cr, Zn and Hg in all sludge samples were detected below 1. CF values < 1 refer to low contamination, 1≤ CF < 3 means moderate contamination, 3 ≤ CF < 6 denotes considerable contamination and CF ≥6 indicates very high contamination. Therefore, all the metals pose low contamination risk, except Mn. The CF value of Mn was higher than 1, indicating a moderate degree of contamination (1 ≤ CF < 3). However, on the basis of mean value of CF, sludge is enriched with metals in the following order: Mn > Zn > Cu > Cr > Cd > Hg > Pb > As. Therefore, Mn and Zn pose maximum enrichment and subsequent contamination to the farm sludge (Table 3).

PLI provides an indication of the sample’s overall toxicity. Moreover, it is also provides useful information to decision makers about the status of the pollution rate of the area [33]. In this study, PLI values in every shrimp farm were found to be below 1, indicating low risk of pollution (Table 3). However, we also analysed PLI, considering the metals that have a high concentration in the sludge, such as Pb, Cr, Mn, Cu and Zn; we found PLI is still below 1 (0.393). Therefore, in both cases, PLI indicates low risk of pollution.

#### Sources of Heavy Metals in Shrimp and Sludge

**Figure 4:**
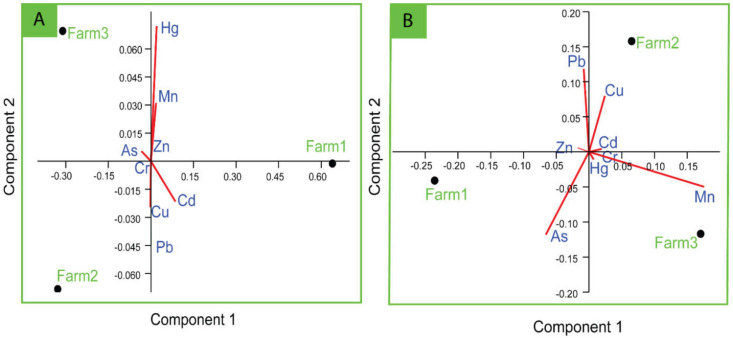
Principal component analysis (PCA) biplot of log-transformed metal concentration in shrimp tissue (**A**) and farm sludge (**B**).

**Figure 5:**
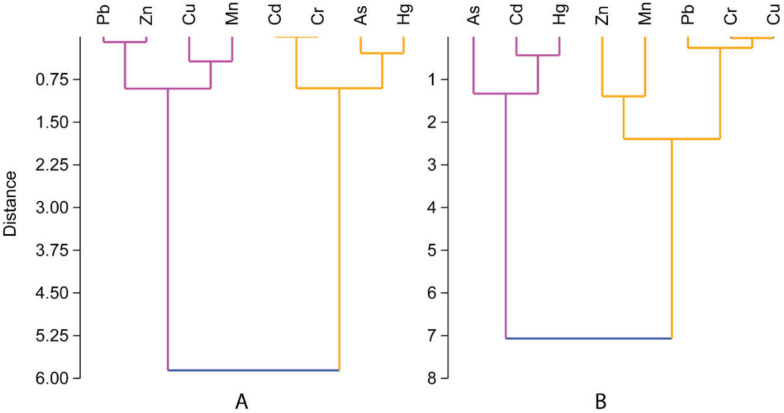
Hierarchical cluster dendrogram (Ward–Linkage method) of metal concentrations obtained from shrimp tissue (**A**) and farm sludge (**B**).

In the aquatic environment, the interrelationship among metals in organisms and sediment provides significant information about sources and pathways of variables (heavy metals). Strong correlations between specific heavy metals may denote similar sorts of contamination and/or release from the same origin of pollution, mutual interrelations and identical behaviour while transporting to the aquatic system [79,80]. However, in our study, in the case of shrimp tissue, we did not observe any linear relationship between the metals obtained. However, in sediment, very strong linear relationships were found between As vs. Cd (0.999) and Cu vs. Cr (0.969) at the significance level 0.05 (Table 4). However, this result motivated us to further analyse the source and association of metals through PCA and cluster analysis. In PCA of metals in shrimp, only two components were able to define all the variance (100%), where the first component described 68.79% of the variance and the second component defined 31.21%. Component 1 was highly loaded with Mn, and component 2 was dominated by Pb, Mn, Cu and As (Figure 4). Similarly, in the HCA of shrimp, two separate clusters were identified, where Pb Zn, Cu and Mn are in a cluster and Cd, Cr, As and Hg are in another cluster. These results of the shrimp tissue samples depict that the shrimp species may uptake heavy metal from different sources, either anthropogenic or natural. Among the metals, the essential trace elements (Cu, Zn and Mn) may be originated from the fish feed [81,82]; therefore, they stand together in the same cluster (Figure 5A). However, in the PCA of farm sludge, component 1 and component 2 described 98.47% and 1.53% of the total variance, respectively. Component 1 was highly loaded with Cd, Mn, As and Hg, whereas component 2 was loaded with Pb, Mn, Cu and Hg. HCA also defined two groups of metals, where As, Cd and Hg are in a group and Zn, Mn, Pb, Cr and Cu made another group. These results indicate that the sources of Cd, As and Hg are the same, whereas the rest might have mutual interrelations. The excessive amount of feed deposition, faeces and dead organisms might increase the concentration of essential trace elements (Cu, Zn and Mn) in the sludge; therefore, their concentration in sludge in our study is higher than that of other metals and their grouping also depicts the same (Figure 5B). However, non-essential metals such, as As, in aquaculture farms could be derived from the groundwater supply [83]. Pb can be introduced from anthropogenic sources, as it is commonly used in the manufacture of batteries, metal products, ammunition and devices to shield X-rays [60]. Other non-essential elements, such as Cd, Cr and Hg, may be introduced through commercialization of shrimp aquaculture systems, drug and chemical uses, improper handling in aquaculture operations and poor waste disposal management.

## 4. Conclusions and Recommendations

This study was undertaken to provide baseline information on the concentrations of some heavy metals in cultured shrimp and aquaculture sludge of coastal areas of Bangladesh. Three shrimp farms were investigated and found to be more or less equally contaminated by some heavy metals. Zinc, lead and copper concentrations were remarkably high in shrimp collected from shrimp farms of Cox’s Bazar, whereas Mn, Zn and Cu were more susceptible to exhibit higher metal accumulation in sludge than shrimp due to its absolute different bioaccumulation pattern. Some metals in shrimp (Pb and Cu) and sludge (Mn, Cr and Zn) exceeded the international quality guidelines. Most of the higher concentrations recovered from shrimp tissue and sludge were of essential trace elements, which might be intensified through food and food residues. The hazard indices showed higher non-carcinogenic health risk for high concentrations of Pb and Cr in shrimp, which is obviously a matter of public health importance for Bangladeshi coastal people that should not be ignored; we should concentrate our efforts to solve this problem with an integrated approach. Thus, continuous monitoring of these toxic metal elements in cultured shrimp and immediate control measures are recommended.

Shrimp is known as the “white gold” of Bangladesh. Shrimp culture and production in Bangladesh has incredibly expanded in recent years, and after fulfilling the country’s demand, a huge proportion of shrimp is exported, which is a major source of foreign currency. However, the increased concentration of heavy metals in shrimp may cause not only human health hazards but also the downfall of the economy. Therefore, to secure the shrimp industry, more intensive research focusing on metals in culture feed, water quality, waste management and handling processes is indispensable. Together with shrimp, our study analysed farm sludge, which was not previously reported in Bangladesh. Therefore, this study may enlighten the stakeholders and management to take necessary measures.

## Figures and Tables

**Table 1:** Comparison of heavy metals (Mean ± SD) in the shrimp muscle tissue samples with various standard levels and relevant study (mg/kg wet weight).

Standard/Study Area	Pb	Cd	Cr	Mn	Cu	As	Hg	Zn	Refs.
Shrimp	17.75 ± 1.5	0.09 ± 0.03	0.69 ± 0.6	4.83 ± 2.2	9.43 ± 2.8	0.04 ± 0.02	0.02 ± 0.006	18.89 ± 2.9	Present study
Tolerance level in crustacean	0.5	0.5	0.5		5	5	0.5	50	[35]
New Zealand	2	1			30	1		40	[36] ^a^
Turkish	1	0.1		20	20			50	[37] ^b^
Institute of Medicine								40	[38]
Bangladesh (crustacean)	0.5	0.5	1		5	5	0.5	50	[39]
River Buriganga, Bangladesh	0.51 ± 0.01	1.51 ± 0.04	1.59 ± 0.9	35.25 ± 1.48	575.34 ± 61.8	1.19 ± 0.04		187.04 ± 9.79	[11]
Saint Martin Island, Bangladesh	0.690 ± 1.56	0.713 ± 0.06	<0.08	<0.2	5.049 ± 0.07	<0.1	<0.03	13.5 ± 0.43	[20]
Sabah, North Borneo	0.38–0.44	0.05	0.04–0.05	0.08–0.11					[40]
Gangetic Delta	9.2	7.7			11.1–48.1			16.1–447.5	[41]
Saudi Arabian Gulf and Jazan, Red Sea, Saudi Arabia	2.33 ± 0.57	1.57 ± 0.066			5.33 ± 0.58			17.33 ± 2.08	[42]

(mg/kg ww) milligrams/kilogram wet weight basis. ^a^ California Environmental Protection Agency, State Water Resources Control. ^b^ Turkish Food Codes.

**Table 2:** Comparison of heavy metals (Mean ± SD) in shrimp aquaculture sludge samples with various standard levels and relevant study (mg/kg wet weight).

Standard/Study Area	Pb	Cd	Cr	Mn	Cu	As	Hg	Zn	Refs.
Sludge	20.23 ± 1.9	0.09 ± 0.2	30.38 ± 2.1	1043.37 ± 59.8	31.14 ± 1.4	0.44 ± 0.34	0.08 ± 0.02	74.72 ± 1.13	Present study
TRV (Toxicity Reference Value)	31	0.6	26		16	6			[62]
LEL (lowest effect level)	31	0.6	26		16	6			[63]
SQG (Sediment Quality Guideline)		6	25					123	[64]
SQG (Sediment Quality Guideline)	40	0.6	25	30				123	[62]
SQG (Sediment Quality Guideline)	35	0.6	37.3					5	[65]
SQG (Sediment Quality Guideline)			0.05	0.5				10	[66]
Tolerance level				123	123	5	10	2	[67]
Halda River	8.80	0.04	8.84	139.5	5.9		0.001	79.58	[68]
Meghna River	6.98	0.53	1.27–6.81						[69]
Sangu River estuary	19.576		25.149		29.235	2.58		261.8	[70]
Korotoa River	36–83	0.26–2.8	55–183		35–118	2.6–52			[71]
Feni River estuary	0.67–17.03		17.77–46.09	23.46–48.73		0.13–2.79	0.87–1.57		[72]
Paira River	25	0.72	45		30	12			[73]

**Table 3:** Environmental health-related hazard indices for metal concentration in sludge.

Metal	Metal Concentration (mg/kg)	Igeo	CF	PLI
Pb	20.23	−0.56	0.1115	0.288
Cd	0.09	−2.32	0.2967	
Cr	30.38	−2.15	0.3376	
Mn	1043.37	−0.371	1.1593	
Cu	31.14	−1.12	0.6920	
As	0.44	−5.44	0.0338	
Hg	0.08	−2.94	0.2000	
Zn	74.72	−0.93	0.7865	

**Table 4:** Pearson correlation analysis of heavy metals in shrimp tissue and farm sludge (*p* values less than 0.05 are indicated as **).

	Pb	Cd	Cr	Mn	Cu	As	Hg	Zn
Shrimp (n = 15, *p* < 0.05 **)								
Pb	1							
Cd	0.088578	1						
Cr	−0.48679	−0.9132	1					
Mn	−0.3571	0.89876	−0.64209	1				
Cu	0.89825	0.51732	−0.82115	0.089732	1			
As	−0.6872	−0.78448	0.96911	−0.43316	−0.93655	1		
Hg	−0.747	0.59604	−0.2171	0.88774	−0.37882	0.030373	1	
Zn	0.25559	−0.94035	0.72009	−0.99431	−0.1953	0.5267	−0.83366	1
Sludge (n = 15, *p* < 0.05 **)								
Pb	1							
Cd	0.10928	1						
Cr	−0.06683	0.98448	1					
Mn	−0.08035	0.98202	0.99991	1				
Cu	0.77784	−0.5397	−0.67904 **	−0.68893	1			
As	−0.1105	−1 **	−0.98427	−0.98178	0.53867	1		
Hg	−0.24948	0.93531	0.98289	0.98529	−0.80265	−0.93488	1	
Zn	−0.90218	0.33019	0.49069	0.50246	−0.97285	−0.32904	0.6428	1

## Data Availability

Data are provided in the article.

## Supplementary Materials

The following supporting information can be downloaded at: https://www.mdpi.com/article/10.3390/toxics10040175/s1, Table S1: Spectral lines used in emission measurements and the instrumental detection limit for the elements measured using AAS; Table S2: Index classification of sediment quality (Muller, 1981).
