# Supplementary material for: Ecological and Human Health Risk Assessment of Heavy Metals in Cultured Shrimp and Aquaculture Sludge
Source: Toxics. 2022 Apr 2;10(4):175. doi: 10.3390/toxics10040175 (PMC9025133; doi:10.3390/toxics10040175)
Supplement: Supplementary file 1 [file toxics-10-00175-s001.zip › toxics-1658101-supplementary.pdf]

toxics

Supplementary Materials: Ecological and Human Health Risk Assessment of Heavy Metals in Cultured Shrimp and Aquaculture Sludge

Salma Sultana, M. Belal Hossain, Tasrina R. Choudhury, Jimmy Yu, Md. Sohel Rana, Md Abu Noman, M. Mozammal Hosen, Bilal Ahamad Paray and Takaomi Arai

Table S1. Spectral lines used in emission measurements and the instrumental detection limit for the elements measured by using AAS.

Elements	Wavelengths (nm)	Instrumental detection limit (mg/l)
Hg	253.7	
As	193.7	
Pb	217.0	0.013
Cr	357.9	0.0054
Cd	228.8	0.0028
Mn	279.5	0.0016
Cu	324.8	0.004
Zn	213.9	0.0033

Table S2. Index classification of sediment quality (Muller, 1981).

I-geo values Muller (1981)	Sediment quality
<0	practically unpolluted
0-1	unpolluted to moderately polluted
1-2	moderately polluted
2-3	moderately to strongly polluted
3-4	strongly polluted
4-5	strongly to extremely polluted
> 5	extremely polluted
**CF values Hakanson (1980)**	
CF <1	refers to low contamination
1≤ CF <3	means moderate contamination
3≤ CF <6	considerable
CF ≥6	indicates very high contamination
